# P2Y1R silencing in Astrocytes Protected Neuroinflammation and Cognitive Decline in a Mouse Model of Alzheimer's Disease

**DOI:** 10.14336/AD.2023.1006

**Published:** 2024-08-01

**Authors:** Shan Luo, Ami Tamada, Yuichi Saikawa, Yifei Wang, Qing Yu, Tatsuhiro Hisatsune

**Affiliations:** Department of Integrated Biosciences, The University of Tokyo, Kashiwa, Chiba 277-8562, Japan.

**Keywords:** Alzheimer's disease, IL-6, P2Y1 receptor, shRNA, AAV-vector, astrocytes, PDGFRβ, Blood-Brain Barrier

## Abstract

Astrocytes, the major non-dividing glial cells in the central nervous system, exhibit hyperactivation in Alzheimer’s disease (AD), leading to neuroinflammation and cognitive impairments. P2Y1-receptor (P2Y1R) in AD brain has been pointed out some contribution to AD pathogenesis, therefore, this study aims to elucidate how astrocytic P2Y1R affects the progression of AD and explore its potential as a new target for AD therapy. In this study, we performed the two-steps verification to assess P2Y1R inhibition in AD progression: P2Y1R-KO AD mice and AD mice treated with astrocyte-specific P2Y1R gene knockdown by using shRNAs for P2Y1R in adeno-associated virus vector. Histochemistry was conducted for the assessment of amyloid-beta accumulation, neuroinflammation and blood brain barrier function. Expression of inflammatory cytokines was evaluated by qPCR after the separation of astrocytes. Cognitive function was assessed through the Morris water maze, Y maze, and contextual fear conditioning tests. P2Y1R inhibition not only by gene knockout but also by astrocyte-specific knockdown reduced amyloid-beta accumulation, glial neuroinflammation, blood brain barrier dysfunction, and cognitive impairment in an AD mice model. Reduced neuroinflammation by astrocytic P2Y1R silencing in AD was further confirmed by the reduction of IL-6 gene expression after the separation of astrocytes from AD mouse brain, which may relate to the amelioration of blood brain barrier as well as cognitive functions. Our results clearly note that P2Y1R in astrocyte contributes to the progression of AD pathology through the acceleration of neuroinflammation, and one-time gene therapy for silencing astrocytic P2Y1R may offer a new therapeutic target for AD.

## INTRODUCTION

The hallmarks of Alzheimer's disease (AD) include amyloid-beta plaque accumulation and neurofibrillary tangles (NFTs) in the brain [1, 2]; however, these two characteristics do not completely define the mechanisms underlying AD progresses. The primary glial cell type in the central nervous system, astrocytes, has long been known to play a crucial role in preserving normal neuronal function [3-6]. In response to CNS injury and pathological processes, astrocytes undergo morphological and phenotypic changes, transforming into a reactive state [7]. These over-activated astrocytes release pro-inflammatory factors that induce inflammation and increase Aβ aggregation, oxidative stress, and glutamate excitotoxicity, thus affecting cognitive function [8-12]. It is therefore crucial to target astrocytes to address pathological processes such as neuroinflammation and to establish reliable therapeutic interventions in AD.

Reactive astrocytes are known to be increased in the AD mouse brain and to be abundantly present around amyloid-beta deposits, while P2Y1 receptor (P2Y1R) is involved in over-activation of astrocytes in AD model mice; thus P2Y1R may correlate with the progression of AD [13]. It has also been demonstrated that administration of MRS2179, a selective inhibitor of P2Y1, to AD mice improved the spatial memory ability of AD mice, probably regulating the calcium activity of astrocytes [14], which may relate to inflammatory responses of reactive astrocytes. However, as a possible effect of P2Y1R in AD, it has also been reported that neural network disorders generated in the CA1 region of the hippocampus, in which overexpression of P2Y1R by calretinin+ (CR) interneurons in late AD leads to increased inhibition of neural networks [15]. In addition, the expression of P2Y1R on a variety of neuronal cells [16-18] leads to the complexity of its effects on AD. Furthermore, recent studies have also shown that P2Y1R exhibit opposite effects on the protection in a variety of pathological settings [19, 20].

In this study, first, we utilized P2Y1R knockout AD mice to confirm the effect of P2Y1R knockout in AD mice, and subsequently set up a miR30-shRNA AAV vector to block P2Y1R expression in astrocytes to test our hypothesis, in which the astrocytic P2Y1 receptor specifically contribute the progression of AD in a mouse model of AD. When the P2Y1 receptor was silenced in astrocytes by one-time gene therapy with AAV vector-based knockdown of target gene expression, we evaluated the number of amyloid-beta plaques loads, as well as cognitive function of an AD model mice.

## MATERIALS AND METHODS

### Mice

In this study, all the mice used were C57BL/6J mice, which were purchased from Sankyo Laboratory Service Corporation (Tokyo, Japan). APP/PS1 mice were purchased from Jackson Laboratory (Bar Harbor, Maine, USA) and mated with B6C3-Tg (APPswe/PSEN1dE9) mice in our breeding room. P2Y1 receptor-deficient mice (hereafter referred to as P2Y1KO mice) were obtained from Prof. Shuichi Koizumi of Yamanashi University. To generate P2Y1 receptor-deficient APP/PS1 mice, P2Y1KO mice were mated with the APP/PS1 mouse line. All animal procedures and experiments in this study were approved by the Ethical Committee of the University of Tokyo and conducted according to the Guidelines for Animal Experimentation of the University of Tokyo, and in compliance with institutional Guide for *the Care and Use of Laboratory Animals*.

### Behavior assessment

Male and female mice at 12 months of age were subjected to behavioral tests, including the 1-day Y-maze test, 3-day contextual fear conditioning test, and 11-day Morris water maze test. Each mouse was transferred to the test room 1 h prior to the test for acclimation. The Y-maze was constructed of dark opaque Plexiglas walls that were 32 cm high, 16 cm wide, and 50 cm long. In each of the three arms, two pairs of infrared photocells were located 21 cm and 42 cm from the arm ends. The customized software recorded the time at the beginning and end of each infrared beam break. Arm entry was recorded when the mice broke the inner arm beam (relative to the center of the maze), followed by the outer arm beam. Arm exits were recorded when the mice broke the outer arm beam followed by the inner arm beam. The three arms were visually identical, so the mice were required to use objects placed in the maze periphery for spatial navigation. Soiled bedding in the maze was stirred between the mice to prevent navigation with odor cues. For each mouse, the three arms of the Y-maze were randomly assigned as the ‘start’, ‘novel’, and ‘other’ arms. Each mouse was placed in the maze for two trials with a 2-h intertrial interval. The ‘novel’ arm was blocked in Trial 1. The mice were placed in the ‘start’ arm and allowed to explore the ‘start’ and ‘other’ arms for 15 min. Following Trial 1, the mice were returned to their home cages. In Trial 2, all three arms were open. Each mouse was placed in the ‘start’ arm and was allowed to explore all three arms for 5 min. The percentage of entries into each arm relative to the total number of arm entries was calculated for each mouse during Trial 2.

For the contextual fear conditioning test, on day 1, a 370 s fear conditioning test was performed on the mice in a box that had been wiped with 70% isopropanol (context A). After placing the mouse in the box, it emitted sounds (85 dB, 5,000 Hz, 10 s) at 128, 212, and 296 s, respectively, and an electric shock (0.75 mA, 1 s) was given to the mouse from the metal wire below, after which the sound was silenced. On day 2, we placed the mice in a box in the same environment (context A) for 512 s, which had been wiped with 70% isopropanol (context A). The box emitted sounds (85 dB, 5,000 Hz, 10 s) at 128, 212, and 296 s without an electric shock. On day 3, the mice were placed in context B for 640 s, which was wiped with 70% ethanol, but the color and shape inside of the box had been changed and was different from context A. Calculate the percentage of freezing time for mice on the second and third day.

We conducted the Morris water maze test in a circular water pool (1.20 m diameter and 45 cm height, temperature 20±2 °C) that was whitened with nontoxic dye. In one quadrant, a white circular platform (10 cm diameter, 35 cm height) was positioned at a depth of 1 cm below the water surface. The Morris water maze task was performed for five consecutive days, followed by a probe trial on the sixth day. Each trial allowed the mice to swim for 60 s to locate the hidden platform and navigate its location using four different spatial clues around the pool. The mouse was allowed to stay on the platform for 10 s once it was found. If the mouse did not find the platform after 60 s, it was guided to it, permitted to stay on top for 10 s, and then returned to the cage. For the reversal learning experiment performed on day 7, the platform was placed in the opposite position from the initial test, and mice were similarly required to find the platform within 60 s. The mouse activities were recorded by a video camera linked to a computer installed with the SMART program (Panlab, Barcelona, Spain) to analyze the time taken to find the platform, measured as escape latency.

### Construction of the targeting astrocyte P2Y1R silencing AAV

To further investigate how P2Y1R influences cognition in AD mice, an AAV vector targeting astrocytes was used for P2Y1R silencing in the mice. A Mammalian Quad miR30-shRNA knockdown AAV vector was used in this study, as described in Fig. 3. The sequences for P2Y1R-shRNAs, which can be seen in Table 1, were screened using the shRNA design website (http://sidirect2.rnai.jp/), as previously reported [21-23], and the matching goal (https://blast.ncbi.nlm.nih.gov/Blast.cgi). The GFAP promoter was used as a specific astrocyte-targeting promoter. The PHP.eB GFAP_pro_-EGFP-P2Y1R-shRNAs vector was produced by standard triple transfection into AAV-pro-HEK293T cells using a pAAV-GFAP_pro_-EGFP-P2Y1R-shRNAs transgene plasmid, pUCmini-iCAP-PHP.eB plasmid, and pAdDeltaF6 helper plasmid. PHP.eB.GFAP.scramble-shRNA.EGFP (VectorBuilder: https://en.vectorbuilder.com/ pDNA (VB210616-1043khj)) served as the control vector.

**Table 1:** Sequences of P2Y1-shRNA.

No.	Site	Sequence
1	911-931	GCATCTCCGTGTACATGTTCA
2	1356-1376	CTTGGGCTGTTATGGATTAAT
3	1465-1485	GCTGTGTCTTATATCCCTTTC
4	1687-1707	AGGAGGAGTGAGGCCAATTTA

### Primary astrocyte culture and transfection

Primary astrocyte cultures were prepared as described by Frangakis and Kimelberg [24]. Briefly, the cerebral hemispheres of newborn C57BL/6J mice were removed, and the meninges were carefully dissected. Cells were grown in DMEM/F12 supplemented with 10% horse serum. The cultures were maintained in a humidified atmosphere containing 95% air/5% CO_2_ at 37 °C. The medium was changed three times per week. After incubation for 8-9 days, the culture bottle was shaken at 240 rpm for 10 h in a constant temperature shaker. The culture solution was then removed, the cells were washed twice with PBS, and 2 ml of 0.25% trypsin digestion solution was added. After digestion at 37 °C for 5 min, 2 ml of DMEM/F12 medium containing 10% FBS was added to stop digestion and then centrifuged at 1000 rpm for 5 min, and the supernatant was discarded. The density was adjusted to 1 by cell counting 10^5^ pieces/ml, inoculated in a poly-L-lysine (PLL)-coated culture bottle, and cultured in an atmosphere of 95% air/5% CO_2_ at 37 °C. Immunofluorescence analyses were performed after three weeks in culture, when the cells formed a confluent monolayer. Immunocytochemically, ≥95% of the cells stained positively for the astrocytic marker GFAP. Transfections were performed as described in the SuperFect™ Transfection Reagent Handbook (QIAGEN, Tokyo, Japan) and were optimized as recommended. For transfection effect studies, 250 μl serum-free medium containing SuperFect Transfection Reagent (25 μl) and 8 μg pGFAP-P2Y1-shRNA plasmid DNA were added to each 100 mm dish. The mixtures were incubated for 15 min at room temperature to allow for the formation of a transfection complex. Subsequently, the mixtures were layered into dishes or wells. The cultures were incubated at 37 °C and 5% CO_2_ for 48 h. The plasmid control group, consisting of the same plasmid without target DNA, and the cell control group (without plasmid DNA) were similarly transfected into astrocytes.

### Intra-cerebro-ventricular injection

Mice were anesthetized with a mixed narcotic solution (0.3 mg/kg medetomidine, 4 mg/kg midazolam, and 5 mg/kg butorphanol tartrate, i.p.). The injection was performed at 10 months of age. Then, each head was positioned in a stereotactic frame, and a midline sagittal incision was made on the scalp. Burr holes were drilled in the skull on both sides over the lateral ventricles using the following coordinates: 0.7 mm posterior to bregma; 1.0 mm lateral to sagittal suture; and 2.0 mm beneath the surface of the brain. The control group received bilateral ICV injections of scramble-shRNA without target DNA silencing effects. Each mouse was injected with 1 μl at each site. The volume of the injection was kept constant, as the variation in the amount of drug in each mouse was minimal, as the mice were in a close weight range. After injection, the mice were kept warm on a 37 °C warm plate until revival.

### Immunofluorescence staining

The mice were sacrificed, and their brains were rapidly removed and merged into a paraformaldehyde (PFA) solution. For immunohistochemistry, frozen specimens were coronally sectioned at a thickness of 40 μm using a cryostat (Microm HM525; Thermo Fisher Scientific, Waltham, MA, USA). After being blocked in 3% donkey serum, the sections were incubated with anti-glial fibrillary acidic protein (GFAP; G3893, mouse, 1:1000; Sigma Aldrich, St. Louis, MO, USA), anti-ionized calcium binding adaptor molecule 1 antibodies (Iba1; 011-27991, goat, 1:1000; Wako; Osaka, Japan), anti-P2Y1 (APR-009, rabbit, 1:200; Alomone Labs, Jerusalem, Israel), anti-PDGFR-beta (AF1042-SP, rabbit, 1:200; Funakoshi, Tokyo, Japan), anti-fibrinogen antibody (ab34269, rabbit, 1:1000; Abcam, Cambridge, UK), anti-lycopersicon esculentum lectin antibodies (DL-1177, mouse, 1:250; Funakoshi), anti-Aβ antibodies (010-26883, mouse, 1:1000; Wako), and anti-GFP (598, rabbit, green fluorescent protein; rabbit, 1:1000; MBL, Tokyo, Japan) overnight at 4 °C. The secondary antibodies were Alexa 488-conjugated donkey anti-mouse IgG (A21202, 1:1000; Invitrogen, Waltham, MA, USA), Alexa 488-conjugated donkey anti-rabbit IgG (A11034, 1:1000; Invitrogen), Alexa 488-conjugated donkey anti-goat IgG (A11055, 1:1000; Invitrogen), Cy^TM^ 3-conjugated affinipure goat anti-mouse IgG (115-165-071, 1:1000; Jackson ImmunoResearch, West Grove, PA, USA) and Alexa 568-conjugated anti-rabbit donkey IgG (A10042, 1:1000; Invitrogen). The stained sections were incubated with DAPI (D9542-10MG, Sigma Aldrich) and observed using a confocal laser microscope (FLUOVIEW FV3000; OLYMPUS, Tokyo, Japan). Images obtained via confocal microscopy were analyzed using ImageJ software.

### Western blot

Western blot analyses were performed as previously described [25]. For brain tissue proteins, the mouse brain tissues were dissected, and the tissues proteins were obtained by using 200 uL RIPA buffer, and a protease inhibitor cocktail was added to prevent protein lysis (RIPA: protease inhibitor cocktail, 100: 1). Cells were cultured in a 6cm dish and collected by scraping. The cell proteins were obtained by 160 uL cell lysis buffer (9803S, Cell Signaling) containing protease inhibitor cocktail (165-26021, Wako). Then, lysates were centrifuged at 15,000 rpm (10,000g) for 10 min at 4°C (for brain, centrifuged 20 min), the supernatants (Triton-soluble fraction) were collected. Proteins were stored at -80 °C until use.

For immunoblotting assays, equal amounts of protein were electrophoresed on SDS-PAGE gel and transferred to an Immobilon polyvinylidene difluoride membrane (ISEQ00010, Sigma). After blocking with 5% milk in Tris-buffered s saline with Tween 20, the membranes were incubated with anti-P2Y1 (APR-009, rabbit, 1:1000; Alomone Labs), and anti-beta actin monoclonal antibodies (66009-1-1G, mouse, 1:5000; Proteintech) overnight at 4 °C. The secondary antibodies used were anti-HRP-conjugated Affinipuregoat anti-mouse IgG (H+L) (U0000326, mouse, 1:5000; Proteintech) and anti-HRP-conjugated Affinipure goat anti-rabbit IgGc (H+L) (U0000438, mouse, 1:5000; Proteintech). Blots were developed by a chemiluminescence reagent (296-69901, ImmunoStar LD, Wako) in an enhanced chemiluminescence (ECL) immunoblotting detection system (LAS-1000, Fuji Film, Tokyo, Japan), and signals were detected using Image Gauge version 3.41 (Fuji Film).

### ELISA

Aβ40 and Aβ42 were measured in protein extracts from both hemispheres using the Human/Rat Amyloid [40/42] ELISA Kit (294-62501, 290-62601, Wako). The standard solution (human amyloid [1-40]) and the standard solution (human amyloid [1-42]) were diluted to the prescribed quantities (100, 50, 25, 10, 5, 2.5, and 1 pmol/l), and the amyloid-beta extraction was diluted 5-fold with diluent and used as the sample. The antigen Aβ11-28 is recognized by BNT77, a monoclonal antibody. Furthermore, BA27, a monoclonal antibody for detecting Aβ40, and BC05, a monoclonal antibody for detecting Aβ42, recognized the edge protein. The next day, the solution was drained from the wells, and the wells were washed five times with the washing solution. After completely removing the washing solution, the wells were filled with 100 µl of HRP-conjugated antibody (BA27) solution or HRP-conjugated antibody (BC05) solution, which was allowed to react with the antigen for 2 h at 4 °C. After the reaction, the solution in the wells was drained, and the wells were cleaned five times with a washing solution. After thoroughly removing the washing solution, 100 µl of substrate solution (tetramethylbenzidine [TMB]) was added to the wells, and the reaction was allowed to proceed at room temperature for 30 min under light shielding. The absorbance at 450 nm was measured using a microplate reader within 30 min after the enzyme reaction was stopped using 100 µl of the reaction stop solution.

### Adult mouse brain dissociation

Mice were perfused transcardially with 1× PBS under deep anesthesia. Brains were then removed, dissected, and rinsed in DPBS; after removing the meninges, a sterile scalpel was used under the microscope to separate the cortex and hippocampus, cut them into small pieces, and centrifuged at 300 × g for 2 minutes at room temperature, and the supernatant was gently aspirated. For enzymatic cell dissociation, the adult brain dissociation kit (130-107-677, Miltenyi Biotec) was used according to the manufacturer's instructions. Place tissue slices (up to 500 mg per slice) in a tube containing 1950 μl of enzyme mixture 1 (enzyme P and buffer Z). Add 30 μl of enzyme mixture 2 (enzyme A and buffer Y) into the tube. Treat with a shaker at 37 °C for 30 minutes. Centrifuge and collect the sample from the bottom of the tube before passing it through a 70 μm filter (130-098-462, Miltenyi Biotec). After that, the cells were washed with D-PBS and centrifuged.

### Magnetic associated cell sorting (MACS) of astrocytes

For isolation of astrocytes, after dissociation of the brain, myelin debris and red blood cells were removed using an adult brain dissociation kit (130-107-677, Miltenyi Biotec). Purified cells were incubated with anti-GLAST (ACSA-1) antibodies to block Fc receptors incubated with anti-GLAST (anti-ACSA-1) MicroBeads (130-095-836, Miltenyi Biotec, Germany) for isolation of astrocytes. ACSA1-positive astrocytes were obtained by magnetic cell sorting with an MS column (921100182, Miltenyi Biotec, Germany). Notably, both the hippocampus and cortex of one mouse were needed to obtain one specimen in this procedure. In this procedure, we can obtain total RNAs for a few numbers of qPCR reaction.

### RNA extraction and quantitative polymerase chain reaction (qPCR)

RNA extraction and qPCR were performed as described by Paredes-Gamero et al. [26] Total RNA was extracted by RNeasy Mini Kit (74104, QIAGEN), and reverse-transcribed into cDNA using SuperScript IV Reverse Transcriptase kit (18090050, Invitrogen). qPCR was performed using the TB Green Premix Ex Taq (RR820A, Takara). The following oligonucleotide primers were used to amplify the nucleotide sequences of P2Y1 receptors: sense primer, 5´ ACCGAGGTGCCTTGGTC GGT 3´ (661-680); anti-sense primer, 5´ CCGGTCTTGG TCAGGGCACA 3´ (800-781) (GeneBank accession no. NM_008772.5). The following oligonucleotide primers were used to amplify the nucleotide sequences of IL-6: sense primer, 5´ GAGCCCACCAAGAACGATAG 3´; anti-sense primer, 5´ TCAGTCCCAAGAAGGCAACT 3´ (GeneBank accession no. NP_112445.1). The following oligonucleotide primers were used to amplify the nucleotide sequences of IL-1β: sense primer, 5´ CA GGCAGGCAGTATCACTCA 3´; anti-sense primer, 5´ TGTCCTCATCCTGGAAGGTC 3´ (GeneBank accession no. NP_032387.1). The primers were purchased from Tsukuba Oligo Service Co., Ltd. The PCR amplification of the nucleotide sequence was performed by incubating the samples at 95 °C for 10 min, followed by 40 cycles at 95 °C for 5 s and 60 °C for 20 s, with a final incubation for 7 min at 72 °C. At the end of amplification, the strands were dissociated.

### Statistical Analysis

Data analyses were performed using SPSS software (version 24.0, SPSS, Chicago, IL, USA), and the GraphPad Prism (v8.0, CA, USA) was employed to plot the diagrams. Data are shown as the mean ± SEM. The two groups were compared using Student’s t test or Mann-Whitney test based on whether the data conforms to the Gaussian distribution. An unpaired t-test with Welch's correction is utilized if both populations do not have equal variances. ANOVA was used to detect significant differences among the three groups followed by Bonferroni’s multiple comparison test. Kruskal-Wallis test followed by Dunn’s multiple comparison test was used to detect significant differences among the three groups if the data does not conform to the Gaussian distribution. All the P-values were two-sided, and the differences were considered statistically significant at P<0.05.

## RESULTS

### P2Y1R knockout resulted in a reduction of amyloid-beta deposits and neuroinflammation

**Figure 1.**
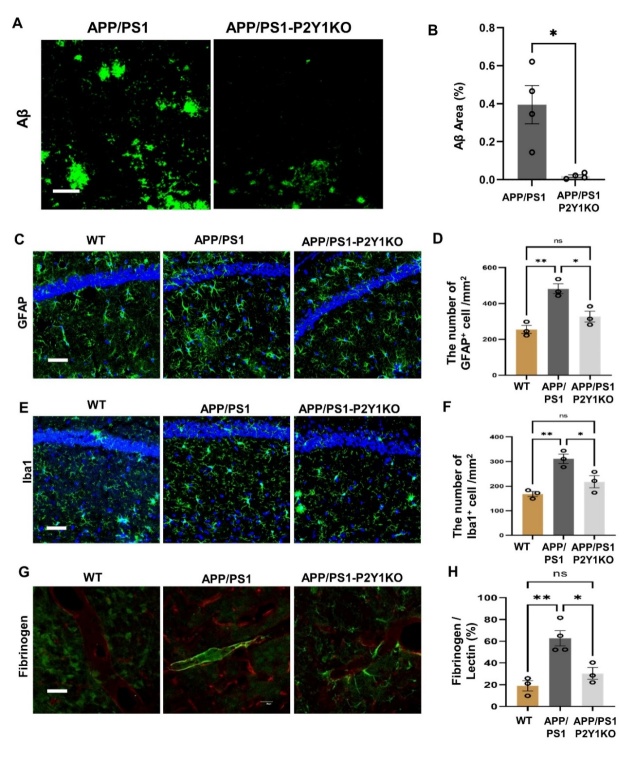
**Knockout of the P2Y1 gene in AD mice reduced amyloid beta protein, inhibited glial activation, and decreased fibrinogen aggregation along the vessel**. (**A**) Typical staining image of amyloid-beta plaques in APP/PS1 (n=4) and APP/PS1-P2Y1KO (n=4) mice (green: amyloid-beta (Aβ) deposits visualized by anti-Aβ immunostaining; scale bar=50 µm). (**B**) Averaged area percentage of amyloid-beta plaque deposits in immune-stained hippocampal slices. The content of amyloid-beta plaques in APP/PS1-P2Y1KO mice was significantly lower than that in APP/PS1 mice. (Mean ± SEM, *p<0.05, Unpaired t-test with Welch’s correction). (**C**) Typical stained image of activated astrocytes in APP/PS1 (n=3), WT (n=3), and APP/PS1-P2Y1R-KO (n=3) mice (green: astrocyte; blue: DAPI; scale bar=50 µm). (**D**) The number of fluorescent-positive (GFAP+) cells was significantly smaller in APP/PS1-P2Y1KO mice than in APP/PS1 mice and was equivalent between APP/PS1-P2Y1KO and WT mice. The microglia from the hippocampus in APP/PS1 (n=3), WT (n=3), and APP/PS1-P2Y1KO (n=3) mice labeled with Iba1 were compared. A classic stained image of APP/PS1, WT, and APP/PS1-P2Y1KO mice (green: microglia; blue: DAPI; scale bar=50 µm). (**F**) The results of immunohistochemical analysis of Iba1+ cells in APP/PS1-P2Y1KO, APP, and WT mice. (**G**) A typical staining image of fibrinogen and blood vessels. (Green: fibrinogen; red: vascular endothelial cells; scale bar=50 µm). (**H**) The proportion of fibrinogen area on vascular endothelial cells in APP/PS1 (n=4), APP/PS1-P2Y1KO (n=3), and WT (n=3) mice was analyzed. The percentage of fibrinogen area on vascular endothelial cells in APP/PS1 mice was significantly higher than that in APP/PS1-P2Y1KO mice. (Mean ± SEM, *p<0.05, **p<0.01, one-way ANOVA test, followed by Bonferroni’s multiple comparison test in D, F and H).

Firstly, the P2Y1R expression in APP/P2Y1KO and APP/PS1 mice were observed by immunostaining, that in the mouse brain of APP/PS1-P2Y1KO, P2Y1R is virtually completely absent (Supplementary Fig. 1). The level of amyloid-beta deposits was evaluated by anti-Amyloid-beta immunostaining. Compared to APP/PS1 mice, P2Y1R-KO AD mice showed less aggregation of amyloid-beta (Fig. 1, A and B). Recent studies have well shown that reactive astrogliosis is a hallmark of AD [27]. We evaluated whether the excessive activation of astrocytes would reduce in APP/PS1-P2Y1R-KO or not. Activated astrocytes were labeled with anti-glial fibrillary acidic protein (GFAP), and their distribution in the hippocampus was assessed with wild type mice served as a control (Fig. 1, C and D). The immunostaining results showed that the number of activated astrocytes (GFAP+ cells) was the highest in APP/PS1 mice, and this overactivation of astrocytes was reduced in APP/PS1-P2Y1KO mice. The number of activated astrocytes was equivalent between APP/PS1-P2Y1R-KO and WT mice (Fig. 1D). Neuroinflammation was further evaluated by the presence of activated microglia that was stained by anti-Iba-1. The number of Iba-1+ cells in APP/PS1-P2Y1R-KO mice was significantly smaller than in APP/PS1 mice and was equivalent to WT mice. In addition, the phenotype of Iba-1+ microglia observed in the APP/PS1 mice had a high-branching type, which was in an activated state, and the cell volume also seemed to be expanded (Fig. 1E). These activated characters were also reduced in APP/PS1-P2Y1R-KO mice similar to WT mice.

**Figure 2.**
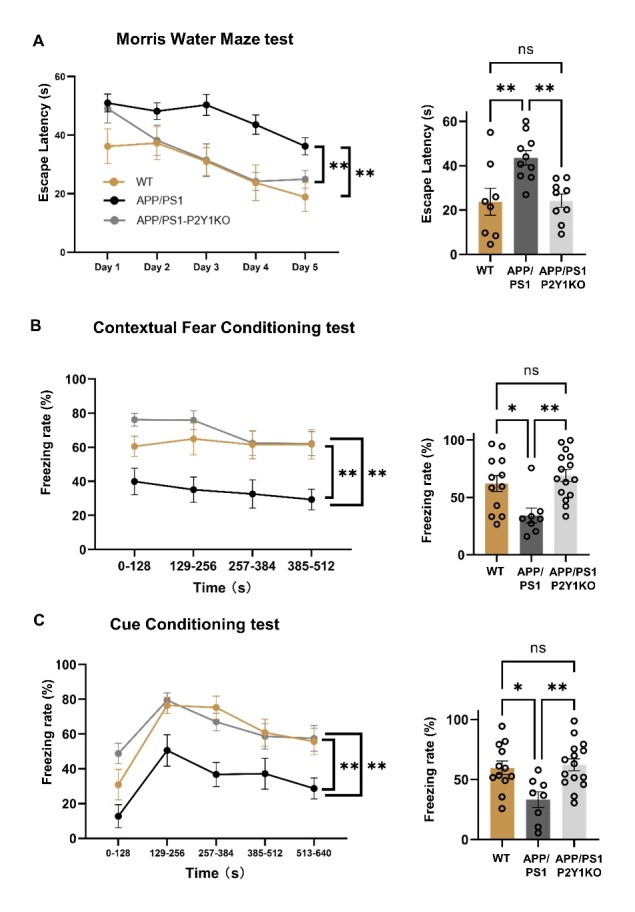
**Results of Morris water maze test (A) and fear conditional tests (B-C) from APP/PS1-P2Y1R-KO, APP/PS1, and WT mice**. (**A**) The 5-day acquisition test of APP/PS1-P2Y1KO (n=9), APP/PS1 (n=10), and WT mice (n=8) was measured by latency time to find a submerged platform under water. For the acquisition test, there were statistically significant differences between the APP/PS1 and APP/PS1-P2Y1KO mice. APP/PS1-P2Y1KO mice discovered the platform significantly quicker than APP/PS1 mice as well as wild type mice on day-4, statistically significant after a post-hoc test. (**B**) The freezing rate of the contextual test on the second day of the CFC test. (**C**) The freezing rate of the cued test on the third day of the CFC test. The results of both the contextual and cued tests between the APP/PS1-P2Y1KO (n=15) and APP/PS1 mice (n=8) approached statistical significance. However, there was no significant difference between the WT (n=12) and APP/PS1-P2Y1KO mice (n=15) in both the contextual and cued tests. (Mean ± SEM, *p < 0.05, **p < 0.01, Line graph: two-way ANOVA test, Bonferroni’s multiple comparison test; Bar graph: one-way ANOVA, Bonferroni’s multiple comparison test).

**Figure 3.**
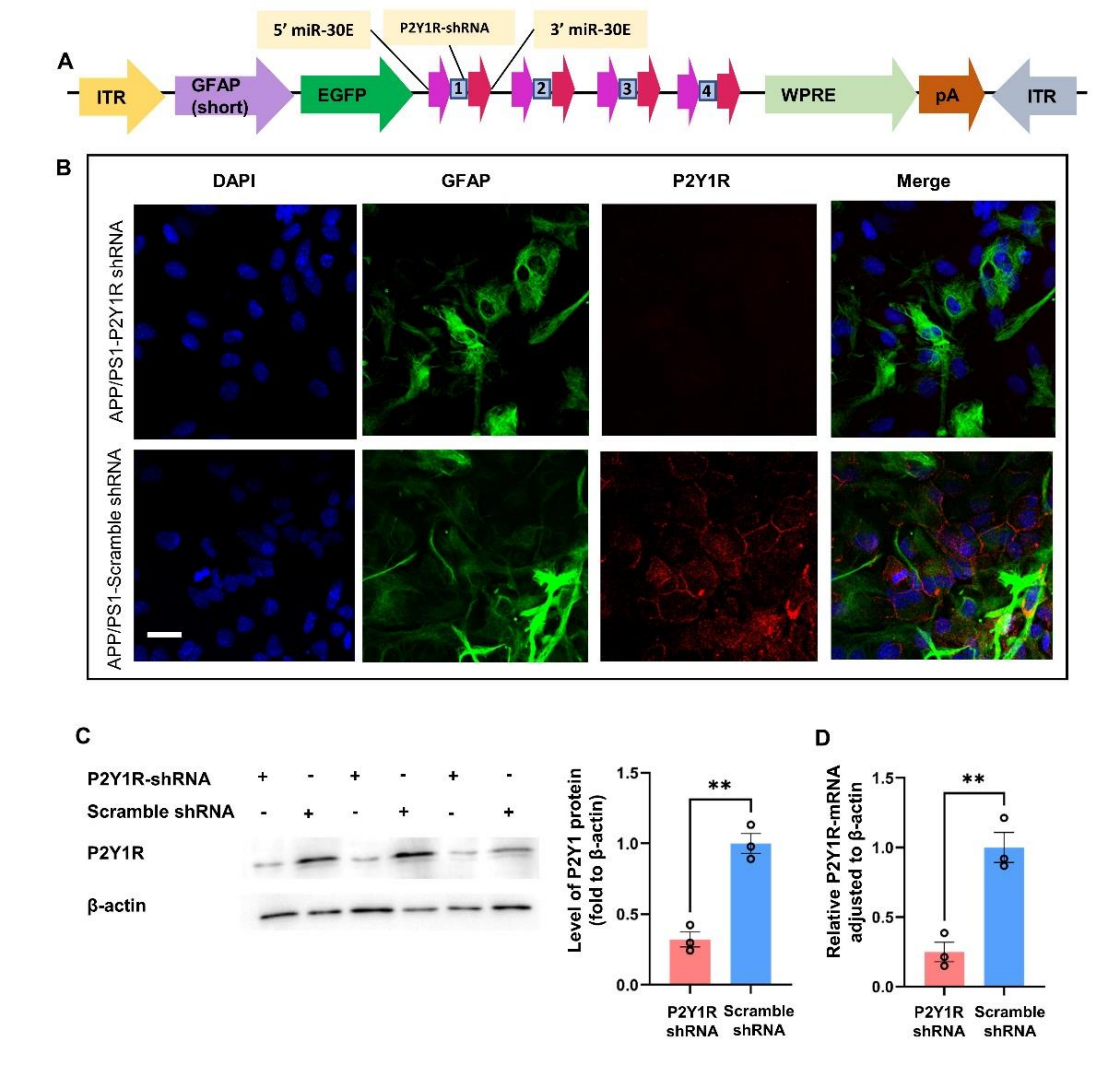
**P2Y1R expression in primary cultured astrocytes treated with P2Y1R-shRNA AAV-vector was measured using immunofluorescence, western blotting, and qPCR**. (**A**) AAV vector MAP used in this study. (**B-D**) Primary astrocytes isolated from mice. (**B**) Typical image of P2Y1R expressed in astrocytes after treatment with shRNA (n=3) in the in vitro cell model (green: GFAP; blue: DAPI; red: P2Y1R; scale bar=50 µm). (**C**) The protein expression of the P2Y1 receptor (~63 kDa); the bar chart shows the presented levels of the P2Y1R in all groups (n=3). Expression of β-actin (~42 kDa) served as a loading control. (**D**) The expression of P2Y1R mRNA in astrocytes in vitro was determined by qPCR. The expression of P2Y1R mRNA in P2Y1R-shRNA-treated astrocytes (n=3) was only 41% of that in the control group (n=3). (Mean ± SEM, **p < 0.01, two-tailed student’s t-test).

### P2Y1R knockout resulted in a reduction of fibrin aggregation on the blood arteries

P2Y1R is often considered to be associated with platelet and blood agglutination in the peripheral system. Studies have shown that P2Y1R is an important receptor that promotes blood agglutination [28]. At the same time, AD is also considered to be a neurodegenerative disease caused by cerebrovascular disease. We aimed to determine whether the effect of the P2Y1R on the improvement of cognitive function in AD mice is related to cerebrovascular improvement. Double staining of vascular endothelial cells (red fluorescence) and fibrinogen (green fluorescence) was used to confirm the effect of the P2Y1R. Aggregation of fibrinogen along the blood vessel wall in APP/PS1 mice was well observed, whereas the aggregation of fibrinogen in APP/PS1-P2Y1KO mice was lower than that in APP/PS1 mice. Moreover, fibrinogen agglutination in the vascular endothelial cells of WT mice was hardly observed (Fig. 1G). At the same time, the ratio of fibrinogen area to vascular area in APP/PS1, WT, and APP/PS1-P2Y1KO mice was determined (Fig. 1H). We can hypothesize that P2Y1R deletion is associated with cerebrovascular integrity, thus improving blood-brain barrier function in AD mice.

**Figure. 4.**
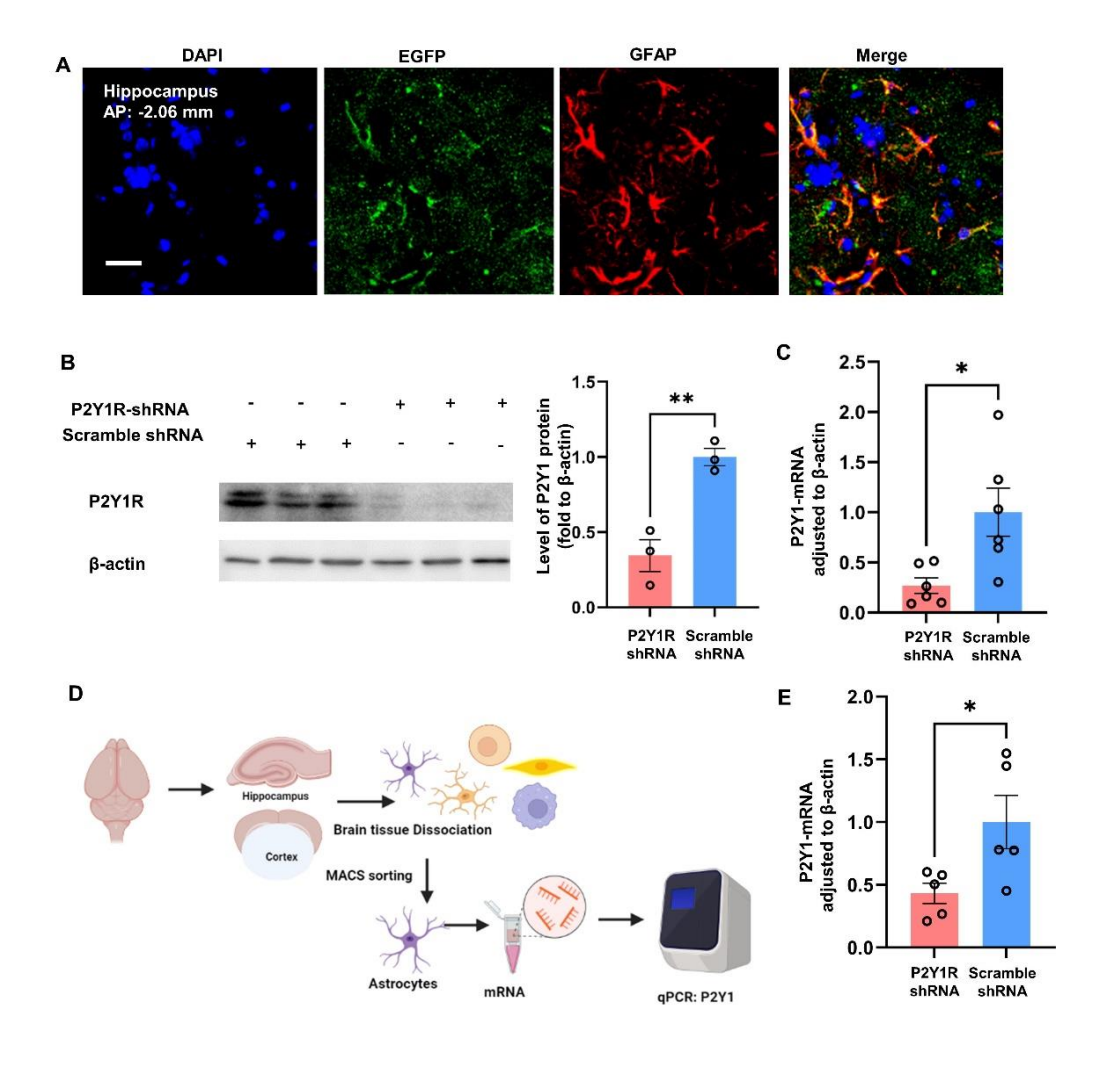
**P2Y1R expression in mouse brain treated with P2Y1R-shRNA was assessed by using immunofluorescence, western blotting, and qPCR**. (**A**) EGFP expression in the brain after P2Y1-shRNA AAV injection. By intracerebroventricular injection, AAV vector can express itself after the intraventricular injection (Green: EGFP; blue: DAPI; red: GFAP; scale bar=50 µm). (B, C) P2Y1R expression in mouse brains after injection with P2Y1R-shRNA AAV vector was determined by western blotting and qPCR. (**B**) The protein expression of the P2Y1R in the brain was assessed by western blotting in the control group (n=3). and P2Y1-shRNA-treated AD mouse group (n=3). (Mean ± SEM, **p < 0.01, two-tailed student’s t-test). (**C**) The expression of P2Y1R mRNA in the brain was determined by qPCR (n=6) (Mean ± SEM, *p<0.05, Unpaired t test with Welch’s correction test). (D, E) P2Y1 mRNA expression in MACS-isolated astrocytes was assessed via qPCR (n=5). (Mean ± SEM, *p < 0.05, Manny-Whitney test).

### P2Y1R knockout resulted in an improvement of spatial and fear memory

To evaluate the effect in spatial memory, 12-month-old APP/PS1 and APP/PS1-P2Y1KO mice were selected as the main experimental subjects, and wild-type mice were added as the control group. From the first to the fifth day of acquisition testing, the time for the APP/PS1 and APP/PS1-P2Y1KO mice to find the platform was significantly shortened with the passage of training time. Compared with APP/PS1 mice, the time for APP/PS1-P2Y1KO mice to reach the platform was shorter (Fig. 2A). In general, APP/PS1 mice took the longest time to reach the platform, and WT mice took the shortest time to reach the platform. There was a significant difference in the time of arrival at the platform between APP/PS1-P2Y1KO and APP/PS1 mice, but there was no significant difference in the time of arrival at the platform between APP/PS1-P2Y1KO and WT mice.

For the contextual fear conditioning (CFC) test, which was used to detect the fear memory of mice, in both the contextual test on the second day of CFC and the cued test on the third day of CFC, the freezing time of APP/PS1-P2Y1KO mice was higher than that of APP/PS1 mice. The freezing time of the WT mice was the shortest. The results of both the contextual test and cued test between the APP/PS1-P2Y1KO mice (n=15) and APP mice (n=8) approached statistical significance. There was no significant difference between WT mice (n=12) and APP/PS1-P2Y1KO mice in both the contextual and cued tests (Fig. 2, B and C).

**Figure 5.**
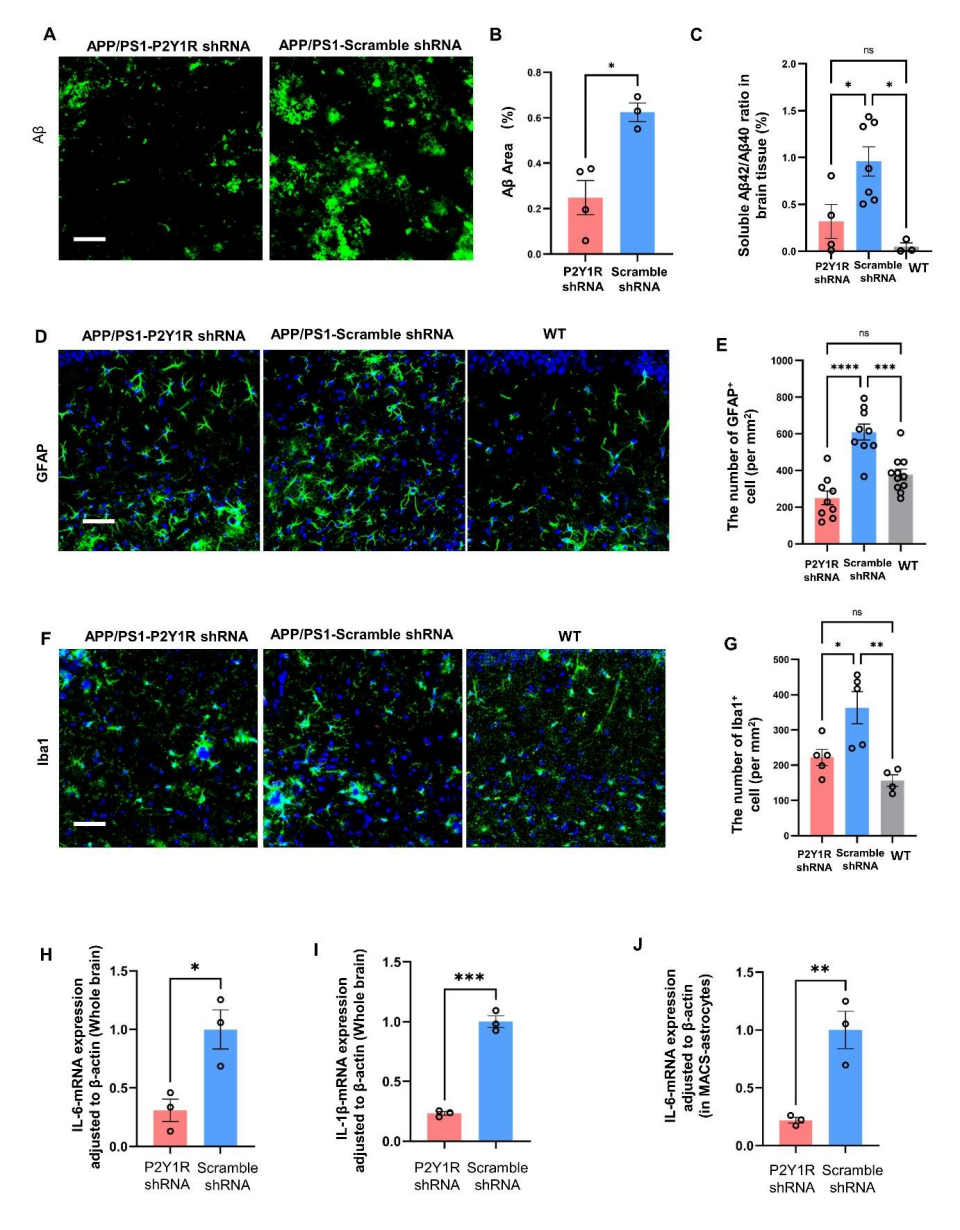
**Astrocyte-specific P2Y1R-knockdown reduced glial neuroinflammation in AD mice**. (**A**) A typical image of amyloid-beta staining. Scale bar=50 μm. (**B**) Statistical results for the amyloid-beta plaque area in immunofluorescence of the control group (n=3) and P2Y1R-shRNA treated AD mice group (n=4). (**C**) ELISA detection of Aβ42 and Aβ40 protein in brain tissue. The ratio of Aβ42 to Aβ40 in the APP/PS1-P2Y1-shRNA group (n=4) and WT group (n=3) was significantly lower than that in the control AAV-treated APP/PS1 group (n=7). (**D**) A typical image of astrocytes. Scale bar=50 μm. (**E**) The number of fluorescent-positive (GFAP+) cells. The number of GFAP+ cells in the APP/PS1-P2Y1R-shRNA group (n=9) and WT group (n=11) was significantly lower than that in the APP/PS1-control-shRNA group (n=9). (**F**) A typical image of microglia. Scale bar=50 μm. (**G**) The number of fluorescent-positive (Iba1+) cells. (H, I) The expression of IL-1β mRNA and IL-6 mRNA was determined by qPCR. The expression of IL-6 mRNA in the APP/PS1-P2Y1 shRNA group (n=3) was significantly lower than that in the control APP/PS1 group (n=3), and there was a significant difference in the expression of IL-1β mRNA between the control APP/PS1 group (n=3) and APP/PS1-P2Y1 shRNA group (n=3). (**J**) The expression of IL-6 mRNA in astrocytes was determined by qPCR. After MACS separation of astrocytes, the expression of IL-6 mRNA in the APP/PS1-P2Y1R shRNA group (n=3) was significantly lower than that in the scramble shRNA (control)-treated APP/PS1 group (n=3) . (Mean ± SEM, *p<0.05, **p<0.01, ***p<0.001, ****p<0.0001; one-way ANOVA test, Bonferroni’s multiple comparison test in C, E and G; two-tailed student’s t test in B, H, I; Unpaired t test with Welch’s correction test in J).

### Targeting reduction of P2Y1R in astrocytes of AD mice

To further determine the role of P2Y1R on astrocytes in metabolic and neurobehavioral control, a miR30-GFAP-P2Y1-shRNA vector was constructed (Fig. 3A); the sequences of P2Y1-shRNA used in this study are listed in Table 1. The efficiency and specificity of the miR30-P2Y1-shRNA adenovirus vector were confirmed using immunofluorescence, western blotting, and RT-PCR in vitro primary cultured astrocytes. P2Y1 was expressed at significantly lower levels in the experimental group than in the control group, as shown by immunofluorescence (Fig. 3B). There was an approximate 67% reduction in P2Y1 protein in P2Y1-shRNA-AAV-treated astrocytes compared with control astrocytes (Fig. 3C). Furthermore, the expression of P2Y1 mRNA in the shRNA-treated astrocyte group was only 41% of that in the control group, which was detected using qPCR (Fig. 3D).

To investigate the role and mechanism of P2Y1R in astrocytes in improving cognitive behavior in AD mice, we generated a model in which the P2Y1 gene in astrocytes was inactivated by delivering the miR30-P2Y1-shRNA adenovirus vector expressing the GFAP promoter-driven into the APP/PS1 mouse brain. At the tissue level, the protein levels of P2Y1R were reduced by 66% in the dissected hemicerebrum, and the expression of P2Y1 mRNA in brain was reduced by 73% in the dissected hemicerebrum (Fig. 4, B and C). The expression of AAV, which has been defined by EGFP, was measured in brain tissue in P2Y1-shRNA-treated AD mice. The AAV can express itself in the cortex and hippocampus by intraventricular injection (Fig. 4A and Supplementary Fig. 2A). After P2Y1-shRNA viral therapy, astrocytes rarely express P2Y1R (Supplementary Fig. 2B). Also, P2Y1 expression in brain astrocytes was analyzed following MACS isolation, showing a 57% reduction with P2Y1-shRNA treatment (Fig. 4, D and E).

### Specific silencing of P2Y1R in astrocytes reduced amyloid-beta deposits and glial neuroinflammation

Amyloid-beta aggregation in the brain is a typical phenomenon of AD; therefore, the level of amyloid-beta aggregation was detected after behavioral testing using immunofluorescence and ELISA. Compared to scramble-AAV-treated AD mice, P2Y1-shRNA-treated AD mice had less amyloid-beta protein in the brain (Fig. 5, A and B). The ratio of Aβ42 to Aβ40 in the brains of P2Y1-shRNA-treated AD mice was significantly lower than that in scramble-AAV-treated (control) AD mice (Fig. 5C). This result indicates that the cognitive ability of AD mice with P2Y1 deletion in astrocytes can be enhanced by changes in amyloid-beta metabolism.

Astrocytes and microglia are important immune cells in the brain, and maintaining the homeostasis of the glial-neuronal network in the brain is important for treating AD. It has been reported previously that P2Y1 receptor blockade normalizes astrocytic hyperactivity [13, 14]. To determine whether P2Y1R in astrocytes contributes to this change, GFAP was used to detect the state of astrocytes. The number of GFAP+ cells in the mouse brain was calculated, and compared to that in APP/PS1 mice, the number of GFAP+ cells in WT and AD mice treated with P2Y1-shRNA was decreased (Fig. 5, D and E).

The microglial numbers in the brains of P2Y1-shRNA-treated AD, WT, and control-AAV-treated AD mice were also calculated by immunofluorescence to further confirm the effect of P2Y1R on glial neural networks (Fig. 5, F and G). The number of Iba1-positive cells in the brains of P2Y1-shRNA-treated AD mice was significantly lower than that in the brains of control-AAV-treated AD mice. As a result, we propose that knocking down the P2Y1 receptor on astrocytes can correct glial cell overactivation in the brains of AD mice, which is beneficial for the brain to maintain the homeostasis of the glial-neuronal network.

**Figure 6.**
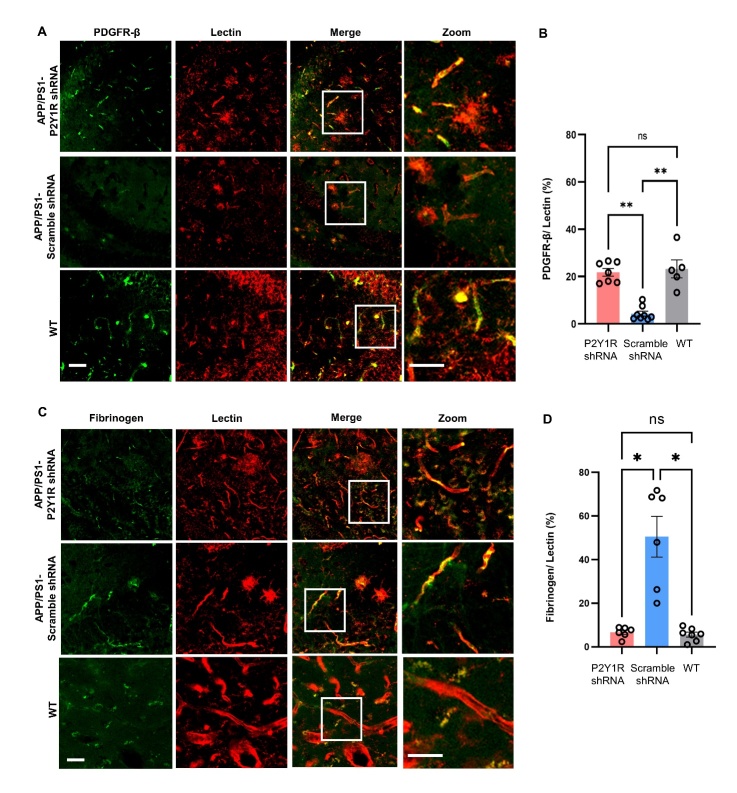
**The expression of PDGFRβ and fibrinogen in brain vessels of P2Y1R-shRNA-treated AD mice, control-AAV-treated AD mice, and WT mice**. (**A**) Typical images of PDGFRβ and lectin in APP/PS1 mice (n=8), P2Y1-shRNA-treated APP/PS1 mice (n=7), and WT mice (n=5). Scale bar=50 μm. (**B**) The ratio of PDGFRβ to lectin in mouse brain tissue. (Mean ± SEM, **p<0.01, Kruskal-Wallis test, Dunn’s multiple comparison test). (**C**) Typical images of fibrinogen and lectin in APP/PS1 mice (n=6), P2Y1-shRNA-treated APP/PS1 mice (n=6), and WT mice (n=7). Scale bar=50 μm. (**D**) The ratio of fibrinogen to lectin in mouse brain tissue. (Mean ± SEM, * p<0.05, one-way ANOVA test, Bonferroni’s multiple comparison test).

To test whether the decrease in the number of overactivated astrocytes would relate to a decrease in the gene expression of inflammatory cytokines, such as IL-1β and IL-6, in the brains of P2Y1-shRNA-treated AD mice, we performed qPCR analyses using primers for IL-1β and IL-6 and observed the reduction of IL-1β and IL-6, significantly (Fig. 5, H and I). Among them, IL-6 is a cytokine synthesized by astrocytes and can induce BBB disorder [29]. We separated astrocytes from AD mice and assessed the change in the gene expression by P2Y1R-shRNA treatment. As a result, we found the reduction in the expression of astrocytic IL-6, as compared to control treatment (Fig. 5J). These results indicate that specific silencing of P2Y1R in astrocytes can reduce the inflammatory response in the brain including brain vasculature and improve cognitive function in AD mice.

**Figure 7.**
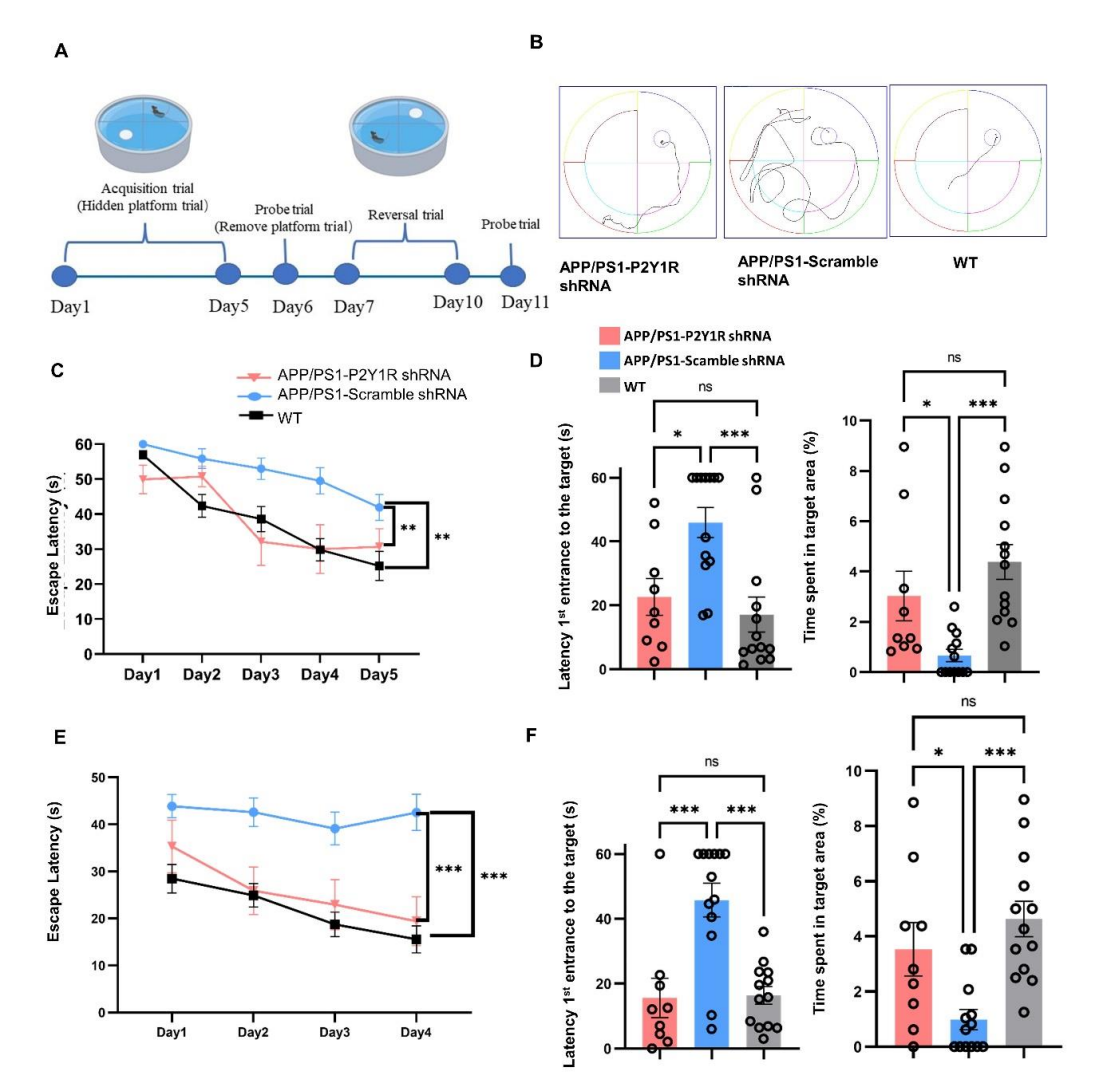
**Morris Water Maze test to evaluate the effect of astrocytic silencing of P2Y1R**. (**A**) The flow tree of the Morris water maze used in this study; (B) representative movement by WT mice (n=13), P2Y1R-shRNA-treated AD mice (n=9), and control-shRNA-treated AD mice (n=13) in the Morris water maze; (C and E) escape latency of the initial test and reversal test; (D) the 1-day probe test after the initial test, in which the platform was removed and measured by the proportion of time spent in the target area and the time mice first reached the target. (**F**) The proportion of time spent in the target area and the time mice first reached the target were measured in the 1-day probe test after the reversal test in which the platform was removed. (Mean ± SEM, *p<0.05, **p<0.01, ***p<0.001, Line graph: two-way ANOVA test, followed by Bonferroni’s multiple comparison test; Bar graph: One-way ANOVA test, followed by Bonferroni’s multiple comparison test).

### Specific silencing of P2Y1R in astrocytes protected BBB function in an AD mice

Reduced PDGFRβ levels mean that pericytes die and blood vessels become vulnerable [30]. Since P2Y1 receptors are primarily associated with platelet agglutination in the peripheral system, we hypothesized that P2Y1 in astrocytes is associated with AD vascular pathology. This means that P2Y1 improves AD cognition by changing the state of astrocytes, thus altering cerebrovascular permeability, and decreasing the expression of intracerebrovascular agglutinogen, thus altering cerebrovascular and cerebral blood flow. To test this hypothesis, immunofluorescence staining was used to examine fibrinogen and PDGFRβ expression in the cerebral vasculature of mice. Compared to the control group, the expression of PDGFRβ in the cerebral vasculature of AD mice with P2Y1-shRNA treatment was significantly increased, while that of WT mice was the highest among the three groups (Fig. 6, A and B). Meanwhile, fibrinogen expression was reduced in the brain vessels of mice injected with P2Y1-shRNA-AAV compared to the control group, and WT was the lowest (Fig. 6, C and D). This finding suggests that blocking the P2Y1 receptor on astrocytes slows vascular damage in AD, which is accomplished through PDGFRβ-mediated changes.

**Figure 8.**
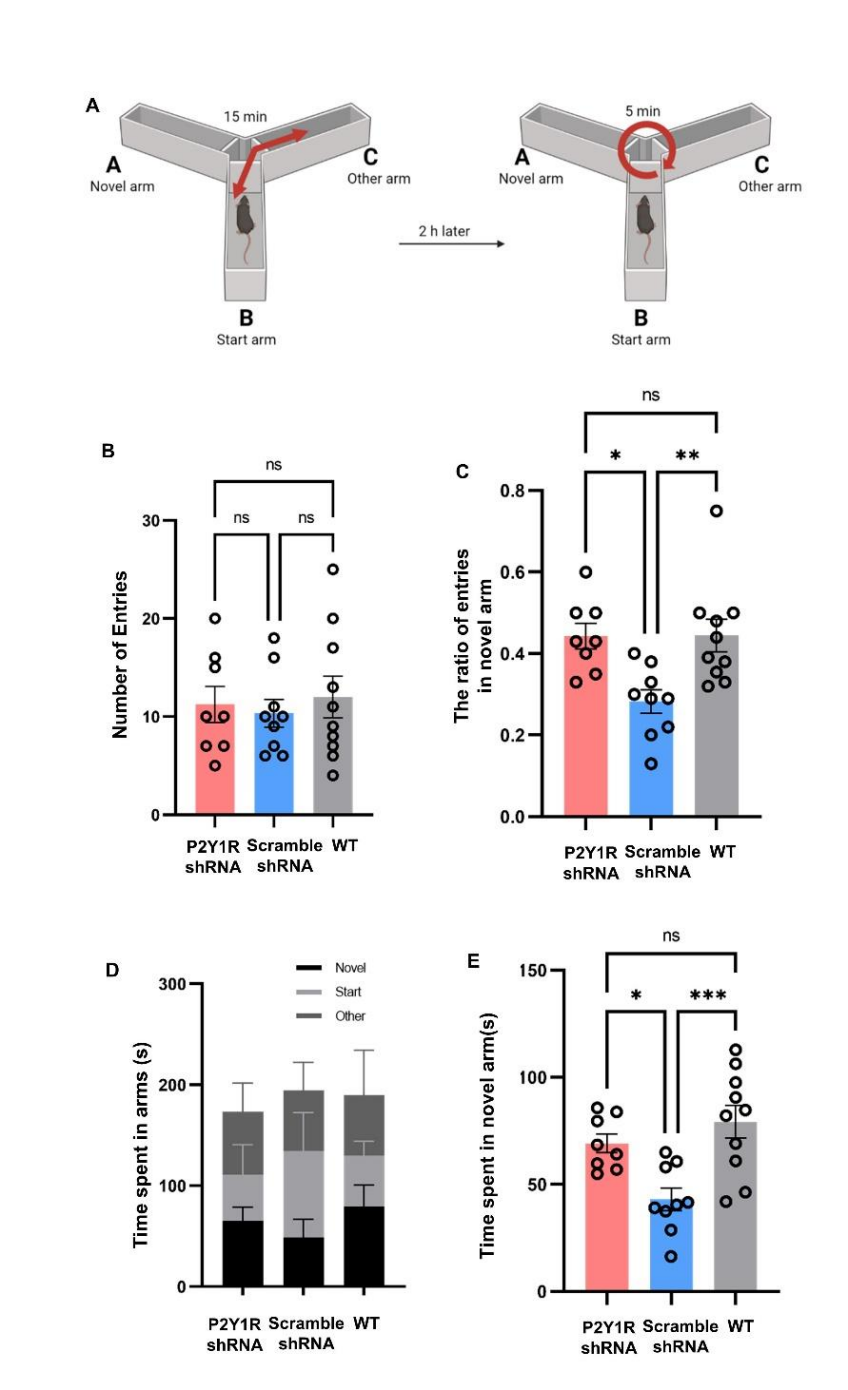
**Y maze test to evaluate the effect of astrocytic silencing of P2Y1R**. (**A**) The flow-process diagram of the Y maze; (B) the total number of arm entries of AD mice injected with P2Y1-shRNA AAV (n=8) or control AAV (n=9) and WT mice (n=10); The number of arm entries did not differ by group; (C) the percent entries of the novel arm; compared with control AAV treated AD mice (n=9), the APP/PS1-P2Y1shRNA mice group (n=8) preferred entry to the novel arm. (D and E) The time mice spent in each arm was calculated. Compared with the control-AAV group (n=9), the WT (n=10) and P2Y1-shRNA-treated AD mouse groups (n=8) spent more time in the novel arm. (Mean ± SEM, *p<0.05, **p<0.01, ***p<0.001, one-way ANOVA test, Bonferroni’s multiple comparison test).

**Figure 9.**
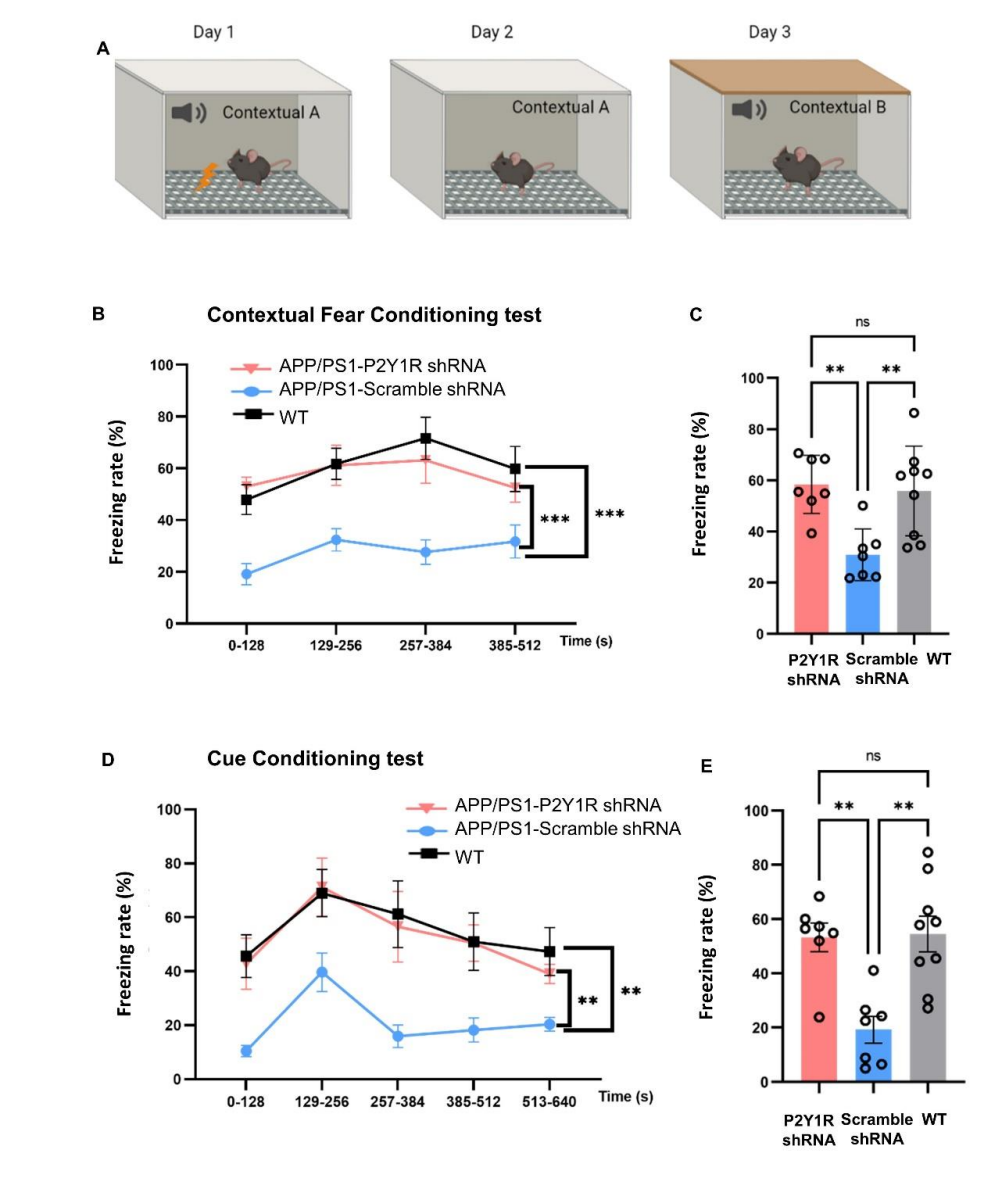
**Fear conditioning test to evaluate the effect of astrocytic silencing of P2Y1R**. (**A**) The schema of the contextual fear conditioning test. (B, C) The freezing rate of the contextual test on day 2 of AD mice injected with P2Y1-shRNA AAV (n=7) or control AAV (n=7) and WT mice (n=9). (D, E) The freezing rate of the cued test on day 3. (Mean ± SEM, **p<0.01, ***p<0.001, Line graph: two-way ANOVA test, Bonferroni’s multiple comparison test; Bar graph: one-way ANOVA test, Bonferroni’s multiple comparison test).

### Specific silencing of P2Y1R in astrocytes ameliorated spatial memory of AD mice

The Morris water maze test was used to assess the spatial memory of the APP/PS1-scramble-AAV, APP/PS1-P2Y1shRNA, and WT mice groups, including a 5-day acquisition test, 1-day probe test, 4-day reversal test, and another 1-day probe test after the reversal test (Fig. 7A). From the first to the fifth day of acquisition testing, the time taken by the mice to reach the platform decreased with time. We found that compared with scramble-AAV group, P2Y1-shRNA-treated APP/PS1 mice had a shorter latency, and WT mice had the shortest latency (Fig. 7C). WT mice and P2Y1-shRNA-treated APP/PS1 mice reached the platform in a shorter path than APP/PS1 mice, indicating that they have a different search strategy (Fig. 7B). In the probe test, in which the platform was removed, the mice in the P2Y1-shRNA-treated group and WT group preferred to stay in the target area compared to the APP/PS1 mice (Fig. 7D). Subsequently, to test the cognitive flexibility in spatial memory, a 4-day reversal test was carried out. In the reversal learning test, the mice in the P2Y1-shRNA-treated APP/PS1 group and WT group reached the platform faster than those in the AD group (Fig. 7E). In a subsequent probe test conducted following the reversal test, P2Y1-shRNA-treated mice spent significantly more time in the target area than the APP/PS1 mice (Fig. 7F). These results suggest that the loss of P2Y1R in astrocytes may help improve the spatial memory of AD mice.

The Y-maze was also used to assess the spatial memory of mice in the APP/PS1-scramble-AAV, APP/PS1-P2Y1shRNA-AAV and WT groups at two months after virus injection; the protocol of this test is shown in Fig. 8A. In this test, activity was measured using arm entries in Trial 2. The percentage of entries into each arm was calculated based on the difference in activity levels over time. Although the total number of entries by WT and P2Y1-shRNA group was similar to that of the scramble-AAV group, the percentage of new arm entries by WT and P2Y1-shRNA mice was higher (Fig. 8, B and C). Compared with scramble-AAV mice, the P2Y1-shRNA group was more likely to enter the novel arm, and the percentage of novel arm entries did significantly differ by group (Fig. 8C). The time spent in the novel arm in Trial 2 was also measured and compared with the scramble-AAV group, the WT and P2Y1-shRNA groups spent more time in the novel arm (Fig. 8, D and E). These data suggest that the spatial working memory of P2Y1-shRNA-treated AD mice was better than that of scramble-AAV-treated AD mice.

### Specific silencing of P2Y1R in astrocytes ameliorated fear memory of AD mice

The contextual fear conditioning test was used to evaluate amygdala-hippocampus-dependent fear memory in the mice (Fig. 9A). In both the contextual test on the second day of CFC and the cued test on the third day of CFC, the freezing time of P2Y1-shRNA-treated AD mice was higher than that of APP/PS1 mice, and the freezing time of mice in the WT group was the longest (Fig. 9, B-E). The results of both the contextual test and cued test between the P2Y1-shRNA treated AD mice (n=7) and APP/PS1 mice (n=7) approached statistical significance, indicating that P2Y1R is related to amygdala-hippocampus-dependent fear memory.

## DISCUSSION

In this study, to elucidate the mechanism of P2Y1R signaling for cognitive decline in AD, we focused on the expression of P2Y1R on astrocytes in the AD brain. It was found that both the knockout of P2Y1R and the knockdown of P2Y1R on astrocytes reduced the number of GFAP- positive and Iba1-positive cells, suggesting that these two modifications for P2Y1R expressions showed equivalent effects on astrocytes which followed by the suppression of neuroinflammatory responses involving over-activation of glial cells and degeneration of BBB function. This finding aligns with our prior study investigating the mechanism through which P2Y1R-KO enhances cognitive function in a mouse model of middle cerebral artery occlusion (MCAO), specifically highlighting the role of glial P2Y1 receptors in modulating the hippocampal inflammatory response [31].

Notably, in this study, we discovered the impact of P2Y1R deletion in astrocytes on BBB function.

Fibrinogen and PDGFRβ were adopted as indicators for the evaluation of BBB function. Among them, fibrinogen is a multipotent protein with an important role in coagulation, inflammation, and tissue repair [32]. Fibrinogen is undetectable in the healthy CNS and accumulates significantly in various neurodegenerative diseases and injuries associated with BBB dysfunction [33]. It has therefore been widely used as a reliable marker of BBB disruption in human tissues and related animal models [34]. In contrast, PDGFRβ is abundantly expressed in pericytes and endothelial cells, and a decrease in PDGFRβ can indicate pericyte death. Pericyte loss or ablation is one of the hallmarks of BBB dysfunction and is thought to trigger a variety of pathological conditions, such as promoting Aβ protein deposition [35, 36]. Our results show that knockdown of P2Y1R on astrocytes resulted in reduced fibrinogen expression and increased PDGFRβ expression, which could indicate restoration of BBB function. Therefore, we suggest that knockdown of the P2Y1R receptor on astrocytes could correct BBB dysfunction in AD. The restoration of BBB function after knockdown of P2Y1R on astrocytes may also be a mechanism for the reduction in Aβ amyloid deposition in the mouse brain.

**Figure 10.**
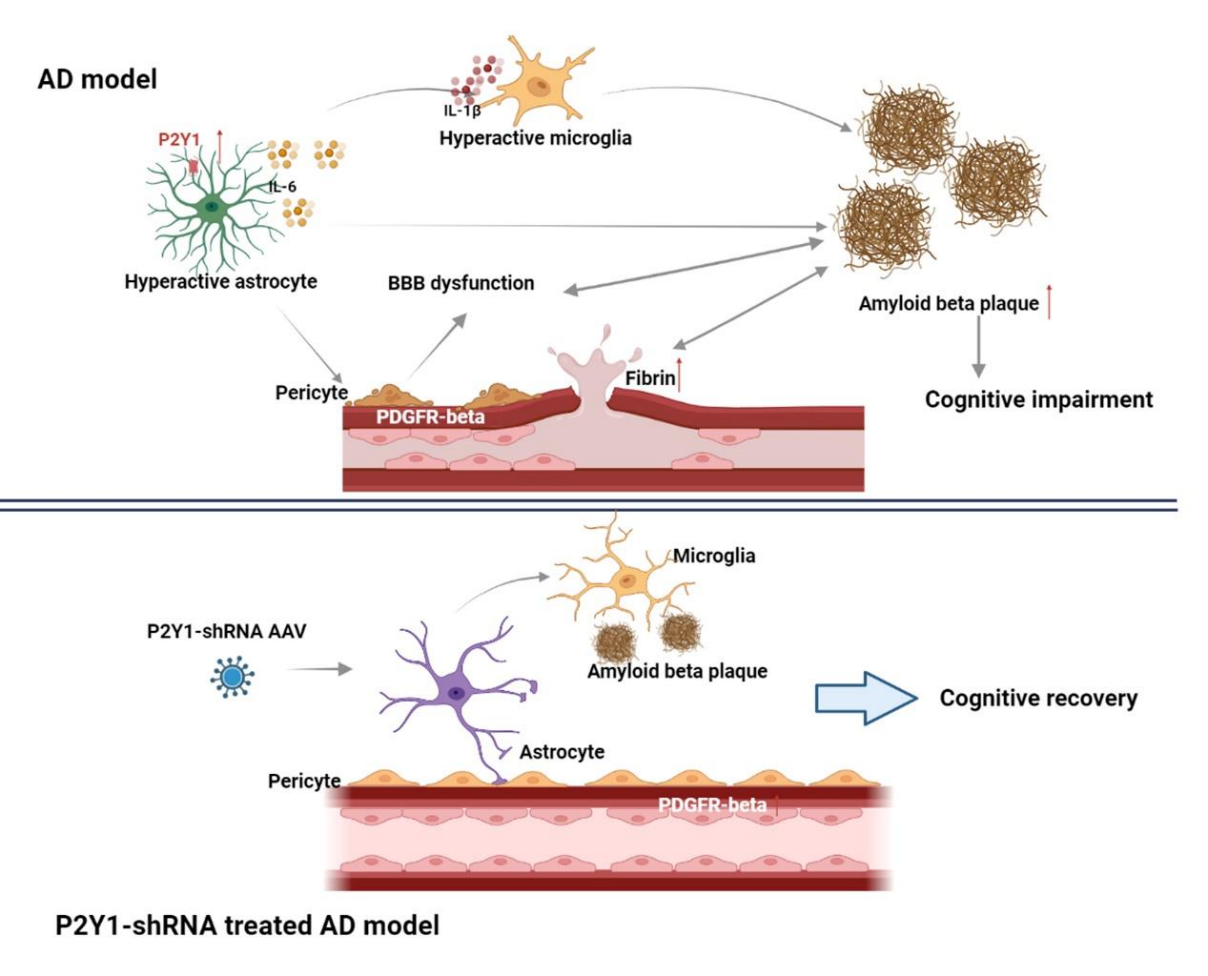
**Schematic illustration of the findings obtained from this study**. Based on this study, we propose how astrocytic silencing of P2Y1R improves cognitive performance in animal model of AD. First, knockdown of the P2Y1R in astrocytes suppresses over-activation of not only astrocytes but also microglia to reduce neuroinflammation, through the reduction of proinflammatory cytokines IL-6 or IL-1β. Second, knockdown of the P2Y1R in astrocytes improve PDGFRβ expression on pericytes and blood-brain barrier function, which may relate to the reduction of amyloid-beta deposition in the brains of AD mice. Third, cognitive impairment in AD mice caused by amyloid-beta depositions, neuroinflammation with excess release of IL-6 and IL-1β, and blood-brain barrier leakage can be reversed by knockdown of the P2Y1R in astrocytes. Therefore, one-time gene therapy for astrocytic P2Y1R silencing can reduce AD symptoms and recover cognitive function of AD mice.

In the present study, knockdown of P2Y1R on astrocytes had an ameliorative effect on cognitive function in AD and could be a potential therapeutic target for AD. Meanwhile, we believe that based on the present study, the main mechanisms of the effect of P2Y1R knockdown on astrocytes on cognitive function in AD are as follows. First, knockdown of the P2Y1 receptor on astrocytes can alleviate overactivation in AD, reducing the overactivated astrocyte production of proinflammatory factors such as IL-6, that involves BBB damage [29] and the deposition of amyloid-beta in AD mouse brains, all of which relate to cognitive decline in AD mice. Second, it is known that the P2Y1 receptor of astrocytes is involved in the activation of microglia. We propose that P2Y1 receptor knockdown in astrocytes affects intracerebral amyloid-beta deposition and that this change results in the restoration of amyloid-beta-disrupted blood-brain barrier function, which in turn results in a decrease in AD cerebrovascular lesions, a decrease in intracerebrovascular fibrinogen expression, and an increase in cerebral blood flow. By further decreasing AD symptoms and enhancing cognitive function in AD mice, this modification further enhances the effectiveness of amyloid-beta transport from the brain to the peripheral system. The mechanism obtained from this study is illustrated in Fig. 10.

In this communication, we have also pointed out the feasibility of astrocyte-targeted shRNA silencing of P2Y1R in clinical AD treatment. A type of gene therapy called shRNA-AAV is a cutting-edge bio-medical technology and is widely utilized as both a research tool and a therapeutic gene therapy technique [37-39]. Regarding the question of whether the shRNA for P2Y1 silencing targeted by astrocytes in this investigation could be employed in clinical settings, we can insist that no adverse effect was observed in AD mice treated with P2Y1-shRNA-AAV in the brain. As delivering shRNA toward brain, we utilized PHP.eB system which can potentially penetrate through Blood Brain Barrier especially in rodents [40], for a future less-invasive shRNA treatments in AD patients. However, at present, there is no such a specific serotype for BBB penetration in primate brain, and it has also been reported that several shRNA-AAVs that are safe in mice unexpectedly showed cerebellar damage when injected into the primate brain [41]. Therefore, before using P2Y1-shRNA-AAV in clinical settings, it is important to generate it from an appropriate adenovirus serotype and utilize it in both small and large animals to monitor for any further negative effects. In addition, the ASL-MRI method established in our laboratory and others can be used to assess the improvement of blood-brain barrier in AD patients treated with P2Y1-shRNA AAV, for the purpose of monitoring the therapeutic effect of P2Y1-shRNA AAV on AD [42, 43]. The results of this study may help in the development of drugs targeting the P2Y1 receptor for the treatment of AD.

In a study targeting the effects and mechanisms of P2Y1R knockdown on astrocytes on AD, we demonstrated that knockdown of P2Y1R on astrocytes can improve cognitive performance in AD mice by reducing the number of reactive glial cells in the brain, slowing down brain inflammation, and improving BBB function. Of note, AD is a chronic and progressive brain disorder that slowly destroys memory, thinking and behavior, although we have identified the 12-month-old APP/PS1-P2Y1KO mice exhibited reduced amyloid beta protein, inhibited glial activation, decreased fibrinogen aggregation along the vessel and better behavior tests, the long-term effects of astrocytic P2Y1R on AD progression remains uncertain. Therefore, longer follow-up is still needed to explore the role of astrocytic P2Y1R on AD, subsequently providing basic evidence for AD therapy.

## Supplementary Materials

The Supplementary data can be found online at: www.aginganddisease.org/EN/10.14336/AD.2023.1006.



## Data Availability

The data supporting the findings of this study are available on request from the corresponding author. The data are not publicly available due to privacy or ethical restrictions.
