# Supplementary material for: P2Y1R silencing in Astrocytes Protected Neuroinflammation and Cognitive Decline in a Mouse Model of Alzheimer's Disease
Source: Aging Dis. 2024 Aug 1;15(4):1969–88. doi: 10.14336/AD.2023.1006 (PMC11272185; doi:10.14336/AD.2023.1006)
Supplement: Supplementary file 1 [file AD-15-4-1969-s.pdf]

SUPPLEMENTARY DATA

P2Y1R silencing in Astrocytes Protected Neuroinflammation and Cognitive Decline in a Mouse Model of Alzheimer's Disease

Shan Luo, Ami Tamada, Yuichi Saikawa, Yifei Wang, Qing Yu, Tatsuhiro Hisatsune*

© 2023. Lou S et al. Published online at http://www.aginganddisease.org/EN/10.14336/AD.2023.1006

SUPPLEMENTARY DATA

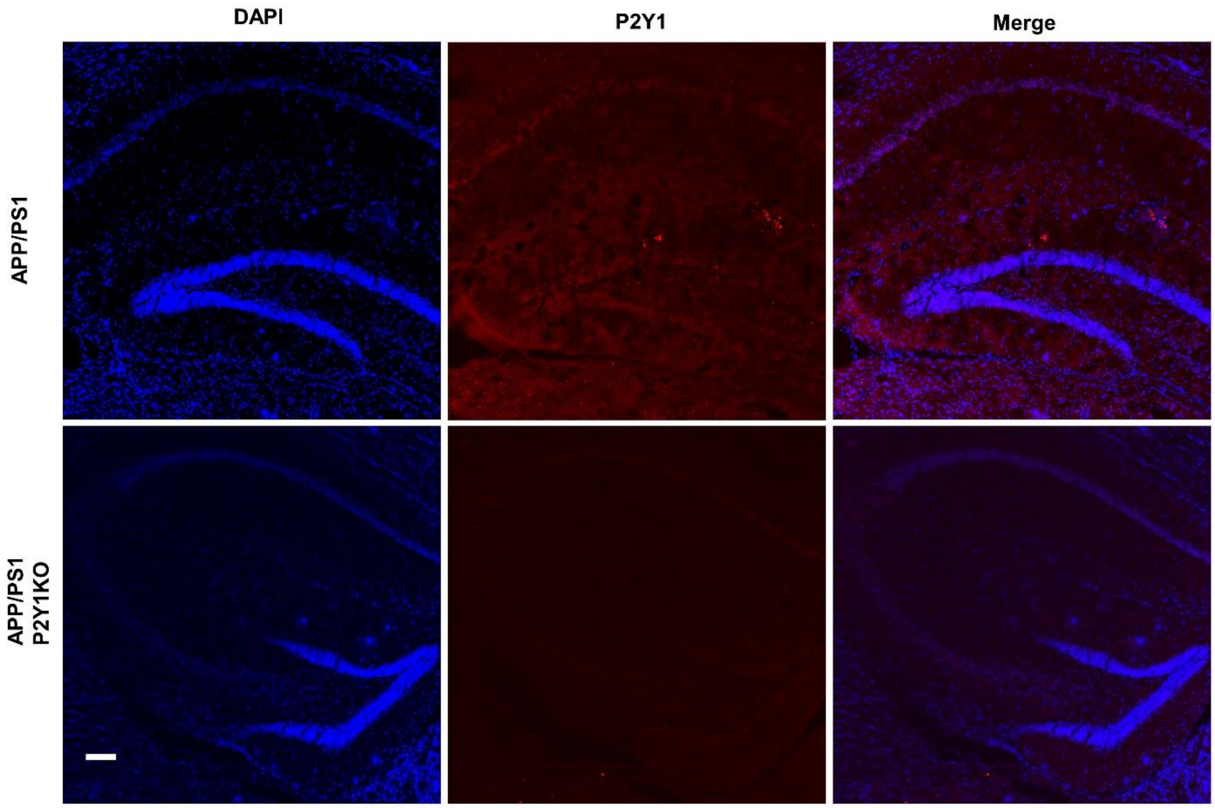

Supplementary Figure 1. The image of P2Y1 receptor in APP/PS1 and APP/PS1-P2Y1KO mice (Blue: DAPI; red: P2Y1R, scale bar = 100 μm). In the mouse brain of APP/PS1-P2Y1KO, P2Y1 is virtually completely absent.

© 2023. Lou S et al. Published online at http://www.aginganddisease.org/EN/10.14336/AD.2023.1006

SUPPLEMENTARY DATA

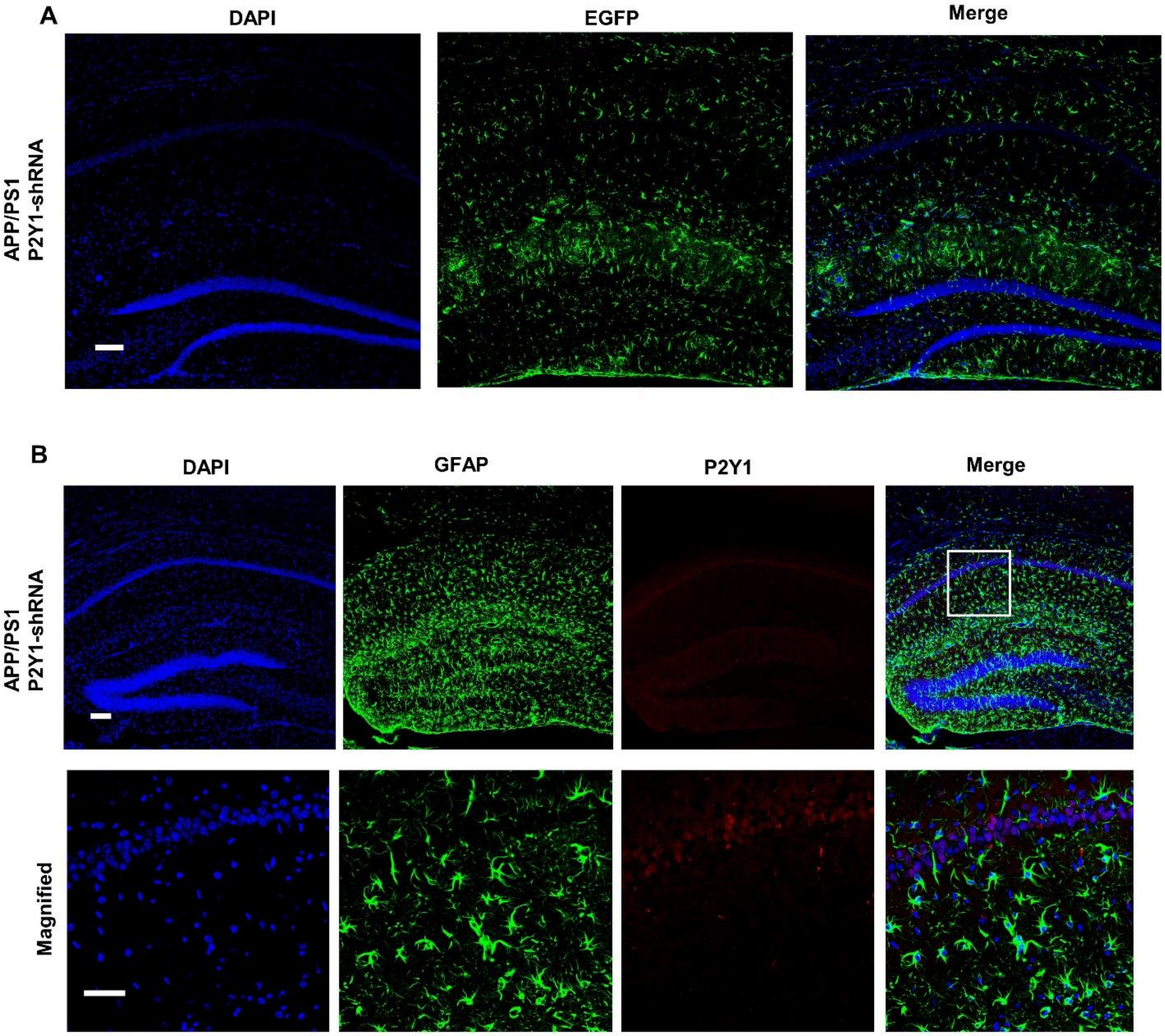

Supplementary Figure2. The image in P2Y1-shRNA treated APP/PS1-P2Y1KO mice. (A) The EGFP expression in hippocampus can be seen after P2Y1-shRNA treated (Blue: DAPI; green: EGFP, scale bar = 100 μm). (B) After P2Y1-shRNA viral therapy, astrocytes nearly never express P2Y1R. (Blue: DAPI; green: GFAP; red: P2Y1R, scale bar = 100 μm; scale bar = 50 μm in magnified figures).

© 2023. Lou S et al. Published online at http://www.aginganddisease.org/EN/10.14336/AD.2023.1006
